# Profiling of coexpression of multiple immune checkpoints in the CD4+ and CD8+ T cell components of the clear cell renal cell carcinoma immune infiltrate

**DOI:** 10.1186/2051-1426-3-S2-P272

**Published:** 2015-11-04

**Authors:** Anne Monette, Nadia Al-Banna, Jean-Pierre Routy, Réjean Lapointe

**Affiliations:** 1 Université de Montréal / Centre de Recherche du Centre Hospitalier de l'Université de Montreal (CRCHUM), Montreal, PQ, Canada; 2 Université de Montréal / McGill University / Centre de Recherche du Centre Hospitalier de l'Université de Montreal (CRCHUM), Montreal, PQ, Canada; 3 McGill University / McGill University Health Centre, Montreal, PQ, Canada

## Introduction

Immune checkpoint blockade therapies have been proven to elicit clinical responses in clear cell renal cell carcinoma (ccRCC) patients. A comprehensive profiling of the tumor landscape is however still required and will help to explain how for this cancer, the high densities of infiltrating CD8^+^ T cells have previously been correlated with a poor clinical outcome. Studies analyzing the tumor immunome often examine gene expression from the complete specimen, which is an overlap of expression profiles from the many different cell types from this microenvironment, and which may make results difficult to interpret. Therefore, we aimed to profile and validate the gene expression signatures from individual CD4^+^ and CD8^+^ immune cell subsets isolated from freshly resected ccRCC patient tumors and blood for their comparison to those from normal kidney tissues and blood, respectively.

## Experimental methods

CD4^+^ and CD8^+^ T lymphocytes were isolated from blood and freshly resected tissues, and RNA was subjected to microarray analysis. Gene expression from a number of resulting targets of interest (immune checkpoints, transcription factors, cytokine/chemokines and receptors, signaling molecules and cytotoxicity markers) were validated using qPCR with the HD Biomark allowing coexpression analysis. Multi-parametric flow cytometry and immunofluorescence were then used to validate the coexpression of immune checkpoint modulators – the coexpression of which was also estimated using survival plots generated from The Cancer Genome Atlas (TCGA) to provide prognostic relevance to our findings.

## Results, conclusions and perspectives

Our findings reveal a differential expression profile of many genes, including immune checkpoint controllers, between the CD4^+^ and CD8^+^ T cells isolated from patient tumors and blood relative to normal kidney tissues and normal donor blood. Our four different sources of T cell isolation allows the discrimination of the differential expression of genes as a function of the disease rather than that of anatomical location. Our work may explain why a high infiltrate of CD8^+^ T cells in ccRCC has been associated to poor clinical outcome, reveal the mechanisms by which this might occur, and provide new strategies for immune checkpoint blockade therapies.

